# Improving accuracy of genomic prediction by genetic architecture based priors in a Bayesian model

**DOI:** 10.1186/s12863-015-0278-9

**Published:** 2015-10-14

**Authors:** Ning Gao, Jiaqi Li, Jinlong He, Guang Xiao, Yuanyu Luo, Hao Zhang, Zanmou Chen, Zhe Zhang

**Affiliations:** National Engineering Research Center for Breeding Swine Industry, Guangdong Provincial Key Lab of Agro-animal Genomics and Molecular Breeding, College of Animal Science, South China Agricultural University, Guangzhou, 510642 China; Department of Animal Sciences, Animal Breeding and Genetics Group, Georg-August-Universität Göttingen, Göttingen, 37075 Germany

**Keywords:** Genomic selection, Bayesian approaches, Priors, Genetic Architecture

## Abstract

**Background:**

In recent years, with the development of high-throughput sequencing technology and the commercial availability of genotyping bead chips, more attention is being directed towards the utilization of abundant genetic markers in animal and plant breeding programs, human disease risk prediction and personal medicine. Several useful approaches to accomplish genomic prediction have been developed and used widely, but still have room for improvement to gain more accuracy. In this study, an improved Bayesian approach, termed BayesBπ, which differs from the original BayesB in priors assigning, is proposed. An effective method for calculating the locus-specific π by converting *p*-values from association between SNPs and traits’ phenotypes is given and systemically validated using a German Holstein dairy cattle population. Furthermore, the new method is applied to a loblolly pine (*Pinus taeda*) dataset.

**Results:**

Compared with the original BayesB, BayesBπ can improve the accuracy of genomic prediction up to 7.62 % for milk fat percentage, a trait which shows a large effect of quantitative trait loci (QTL). For milk yield, which is controlled by small to moderate effect genes, the accuracy of genomic prediction can be improved up to 4.94 %. For somatic cell score, of which no large effect QTL has been reported, GBLUP performs better than Bayesian methods. BayesBπ outperforms BayesCπ in 10 out of 12 scenarios in the dairy cattle population, especially in small to moderate population sizes where accuracy of BayesCπ are dramatically low. Results of the loblolly pine dataset show that BayesBπ outperforms BayesB in 14 out of 17 traits and BayesCπ in 8 out of 17 traits, respectively.

**Conclusions:**

For traits controlled by large effect genes, BayesBπ can improve the accuracy of genomic prediction and unbiasedness of BayesB in moderate size populations. Knowledge of traits’ genetic architectures can be integrated into practices of genomic prediction by assigning locus-specific priors to markers, which will help Bayesian approaches perform better in variable selection and marker effects shrinkage.

**Electronic supplementary material:**

The online version of this article (doi:10.1186/s12863-015-0278-9) contains supplementary material, which is available to authorized users.

## Background

In the field of medicine, risk prediction of major diseases such as cancer is essential for taking preventive measures early before worsening [1–4]. Similarly, it is important to predict genetic values of candidates for early selection, through which the production costs will be reduced immensely, in breeding programs both of domestic animal and economically important plants [5–9]. Therefore, developing prediction methods exploiting the availability of genomic big-data is a renewed hot topic in the scientific community nowadays.

With the development of high-throughput sequencing technology and the commercial availability of genotyping bead chips in recent years, large numbers of single nucleotide polymorphisms (SNPs) covering the whole genome can be obtained quickly and cheaply. The utilizations of these genomic data to accelerate genome wide association studies (GWAS), disease prediction and personal medicine of human beings, and breeding programs of animals and plants are attracting more and more attention [10]. The paradigm of involving dense genomic markers into genetic merit prediction, which was termed genomic selection (GS), was first proposed by Meuwissen et al. [11]. Nowadays, GS has been applied to genetic merit prediction in human beings [12], model organisms [13], dairy cattle [14–17] and other domestic animals [18–21], and has even been applied to the breeding programs of economically important crops [22–24], forest trees [25, 26], and aquaculture species [27]. Methods for GS keep developing rapidly [28] and can be divided into two categories, direct and indirect approaches, based on the manners in which they use the genetic markers [29]. Direct approaches are derived from best linear unbiased prediction (BLUP), and termed genomic best linear unbiased prediction (GBLUP) [30], which firstly construct a numerator relationship matrix with SNPs and then mixed model equations are solved to obtain genetic merit directly. Indirect approaches include ridge regression BLUP (RRBLUP) [11], Bayesian variable selection approaches [11, 31], and Bayesian shrinkage [32, 33] first estimate marker effects and then get genetic values by summing the effects of all relevant markers.

In Meuwissen et al. [11], least square (LS), RRBLUP, and Bayesian non-linear models (i.e., BayesA and BayesB) were compared for the accuracy of quantitative trait loci (QTL) detection and genetic value prediction. Results from literatures shown that Bayesian approaches, which integrate a priori that a large proportion of SNPs (with a high probability *π*) are non-effective, are more powerful than other models in both of QTL detection and genetic value prediction. Since 2001, many Bayesian methods for GS have been developed [11, 31, 34], which were reviewed by Meuwissen [28]. The concept of a “Bayesian alphabet”, which denotes the growing number of Bayesian methods that differ in the priors while sharing a similar sampling model, was first proposed by Gianola [35].

Although widely used in the animal and plant breeding programs, the original Bayesian models have been shown to have some drawbacks [31, 35]. The first is the arbitrary assignment of the proportion of non-effective SNPs (*π*), which is treated as a constant close to 1 (i.e., 0.95 or 0.99) in most situations [31, 35]. The second is the data-independent prior degree of freedoms assigned to locus-specific variances [35]; The full-conditional posterior has only one additional degree of freedom compared to the prior distribution, regardless of the number of phenotypes and genotypes [31]. To overcome these two deficiencies, BayesCπ and BayesDπ [31] were developed, in both of which the non-informative parameter *π* and/or scale parameter *S* are treated as variable and sampled from relevant prior distributions. Additionally, changes to the distribution of marker effects and variances have been performed [36, 37]. BayesLASSO [38, 39] uses an exponential distribution as a prior of marker effects, different from the prior normal distribution in BayesA and BayesB. In BayesR [15] and BayesRS [34], the prior of marker effects is treated as a serious normal distribution. All of these approaches show some advantages under different circumstances, but none of them can be considered as the golden rule.

Although Bayesian approaches outperform GBLUP under most circumstances, the priors assigned to the established Bayesian approaches still may have room for further improvement. It has been shown that genetic architectures of traits can influence genomic prediction accuracy [40]. Therefore, traits’ genetic architectures should be taken into account by assigning locus- or trait-specific priors to genomic prediction models. By assigning different marker weights to build a trait-specific numerator relationship matrix, locus-specific priors have been utilized in methods derived from BLUP, such as TABLUP [41, 42], BLUP|GA [43], and iterated-GBLUP [44]. These approaches confirmed that locus-specific priors show benefits compared to common priors. Moreover, by converting *p*-values derived from GWAS into marker-specific weights, the locus-specific priors have been utilized in the genomic prediction of human traits via BLUP [12], through which a greater degree of accuracy was gained. All these previous studies indicated that locus-specific priors in genomic prediction show favorable features in BLUP models. However, it has not been tested whether more accuracy will be gained in Bayesian models with locus-specific priors. Based on the assumptions of BayesB and prior knowledge of traits’ genetic architectures, we argue here that a locus-specific prior (π) is more appropriate for Bayesian methods for genomic prediction. With a locus-specific prior, the accuracy of genomic prediction may be improved due to a more appropriate marker effect shrinkage and variable selection. The aim of this study is to propose and validate a modified BayesB method which can utilize locus-specific priors. The performance of the modified Bayesian approach in genomic prediction is compared with that of GBLUP, the original BayesB and BayesCπ.

## Results

### Statistical summary for all traits

Two datasets, a German cattle population [45] and a loblolly pine (*Pinus taeda*) dataset [25] were analyzed in this study. The statistical summary of all traits in the two datasets are shown in Table 1. It should be noted that phenotypes in the German cattle population were rescaled to standard normal distribution, i.e., *y* ~ *N*(0, 1), where y denotes the phenotypes. For these traits, the traditional estimated breeding values, with high reliability, were close to the true breeding values. The variation of the regressed phenotypes of the loblolly pine was dramatically large (Table 1), and their heritability are relatively low [25].

**Table 1:** Descriptive statistics of trait phenotypes

Datasets	Traits^a^	N	Min.	Mean	Max.	S.D.	CV%
Dairy cattle	MY	5024	−3.383	0.000	3.319	1.000	–
	MFP	5024	−3.569	0.000	4.281	1.000	–
	SCS	5024	−4.462	0.000	3.469	1.000	–
Loblolly Pine	HT	927	−287.700	20.300	226.10	73.315	361.158
	HTLC	927	−94.110	3.304	89.080	24.976	755.932
	BHLC	927	−1.578	0.092	1.573	0.507	551.087
	DBH	927	−5.439	0.294	1.349	4.150	1411.565
	CWAL	927	−91.190	2.443	130.800	27.326	1118.543
	CWAC	927	−140.600	2.276	157.000	42.033	1846.793
	BD	927	−0.608	−0.004	1.739	0.249	−6225.000
	BA	927	−24.560	−0.261	21.140	7.315	−2802.682
	Rootnum_bin	927	−0.779	0.107	0.602	0.258	241.121
	Rootnum	927	−2.422	0.321	4.368	0.960	299.065
	Rust_bin	927	−0.482	−0.014	0.822	0.399	−2850.000
	Rust_gall_vol	927	−1.175	−0.022	5.212	1.132	−5145.454
	Stiffness	927	−3.244	0.095	6.082	1.225	1289.474
	Lignin	927	−3.644	0.050	4.073	1.200	2400.000
	LateWood	927	−4.544	0.090	4.878	1.571	1745.556
	Density	927	−10.290	−0.053	17.610	2.498	−4713.208
	C5C6	927	−8.102	−0.049	9.057	2.649	−5406.122

^a^MY, milk yield; MFP, milk fat percentage; SCS, somatic cell score; HT, total stem height; HTLC, total height to the base of the live crown; BHLC, basal height of the live crown; DBH, traits stem diameter; CWAL, crown width along the planting beds; CWAC, crown width across the planting beds; BD, average branch diameter; BA, branch angle average; Rootnum_bin, presence or absence of roots; Rootnum, Root number; Rust_bin, presence or absence of rust; Rust_gall_vol, gall volume; lignin, lignin content; LateWood, latewood percentage; Density, wood specific gravity; C5C6, C5C6 content. In the dairy cattle population, phenotypes were rescaled to standard normal distributions

### Capturing of genetic architecture

**Fig. 1:**
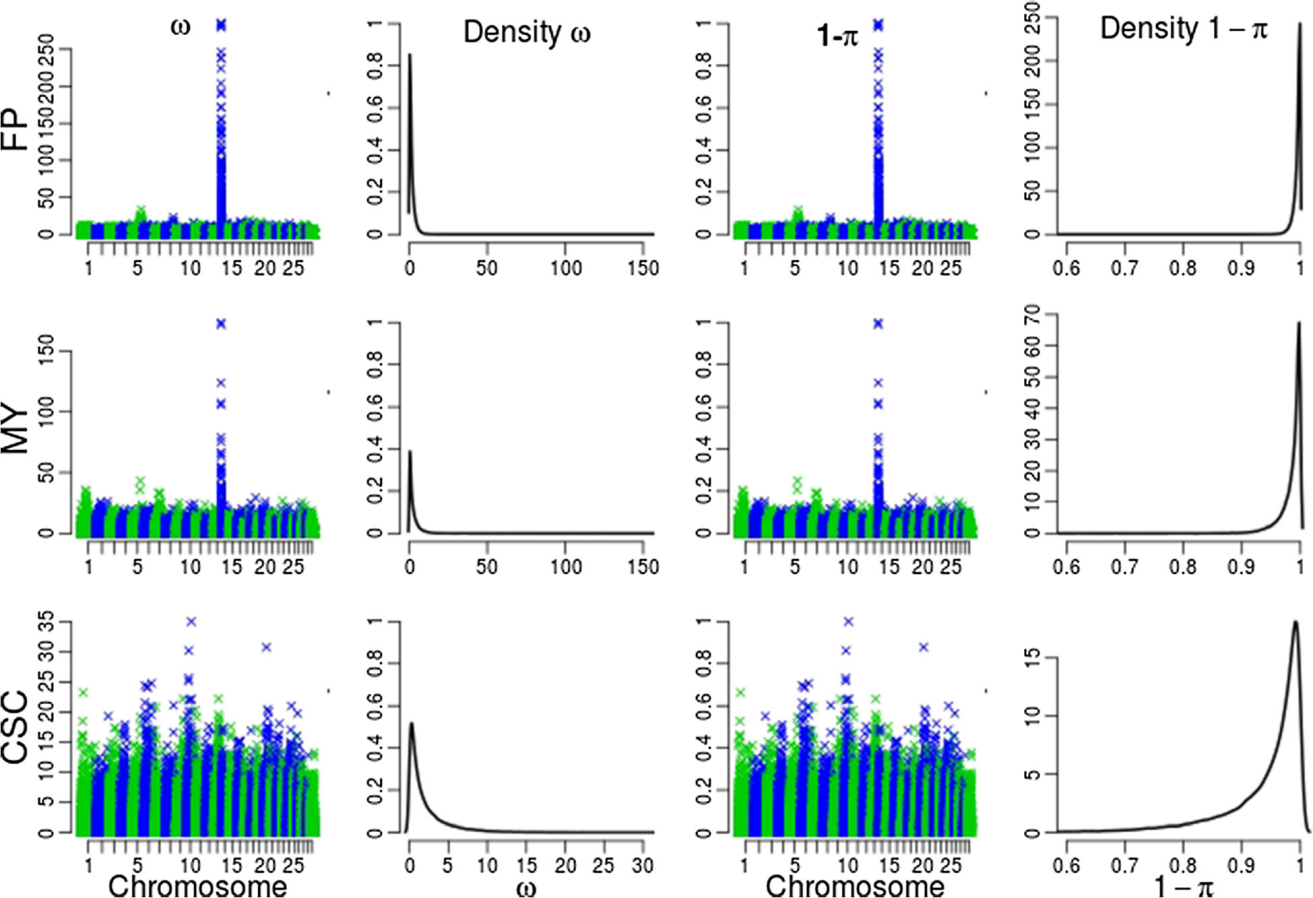
Distribution of *p*-values and locus-specific π of three traits in dairy cattle population across the genome. *Rows* in the figure correspond to distributions of features of milk fat percentage (FP), milk yield (MY), and somatic cell score (SCS), respectively. Four columns correspond to distributions of *ω*, density of *ω*, distribution of locus-specific π, and density of locus-specific π, respectively; where, *ω* = − *log*_10_(*p* − values). The *p*-values are derived from ANOVA for all single markers. Logarithmic transformation of the *p*-values is performed for data visualization convenience and latter utilization. The locus-specific π is derived from the *p*-values of the ANOVA via formula (4). Since π is the proportion of non-effective markers, 1-π is taken as the probability of each marker to be effective. For milk yield and milk fat percentage, the clusters on chromosome 14 is the genomic segment where located the *DGAT1* gene. For somatic cell score, no cluster is observed due to the lack of major genes. Distributions of the locus-specific π are consistent with our prior knowledge about the genetic architectures of these traits. These plots are drawn on the R software platform (http://www.r-project.org/)

In order to capture genetic architectures of traits, analysis of variance (ANOVA) based on single markers is performed for three traits in the dairy cattle population. The logarithms of *p*-values from ANOVA reflect the genetic architecture of these traits (Fig. 1). For milk fat percentage, a set of SNPs with dramatically low *p*-values on chromosome 14 were detected via ANOVA (Fig. 1), which is consistent with our prior knowledge about the genetic architecture of this trait that 30 % of the genetic variation is due to segregation of the *DGAT1* gene [46, 47] located on chromosome 14. For milk yield, clusters of SNPs with low *p*-values were detected on chromosome 1, 5, 7, and 14, which is consistent with the prior knowledge that there is a major gene on chromosome 14 and some genes with moderate or small effects on other chromosomes. For the somatic cell score, none significant association between phenotypes and SNPs has been detected, which in agree with the prior knowledge that no major genes affect this trait.

Furthermore, we found that the *p*-values from ANOVA can be converted with formula (4) to a probability form, which can be used as a genetic-architecture-based *π* in Bayesian methods. The distribution of locus-specific *π* for three traits in the dairy cattle population can reveal the genetic architecture of these traits at some extent (Fig. 1).

### Validating BayesBπ with the German dairy cattle dataset

**Fig. 2:**
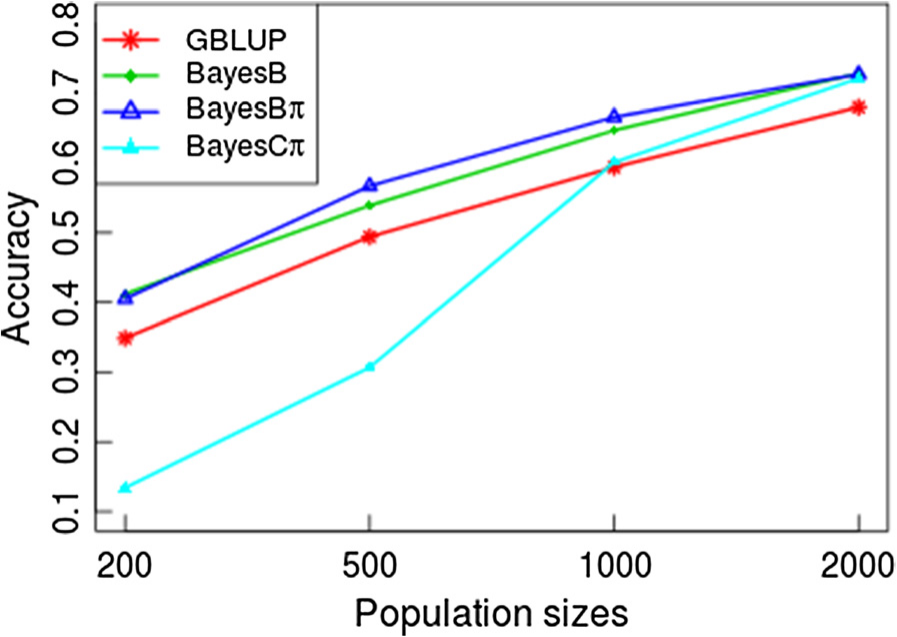
Impact of population sizes on genomic prediction accuracy. Genomic prediction accuracies of each method in each subpopulation are averaged among three traits to test the overall performance of methods in different subpopulations. For example, accuracies of GBLUP in subpopulation 200 are averaged among three traits to gain its’ overall performance in this population size

Results of genomic prediction for three traits in the German Holstein dairy cattle (Table 2) show that when the population size is 200, BayesB outperforms BayesBπ. However, when the population sizes are 500 and 1000, BayesBπ performs better than BayesB. For milk fat percentage, BayesBπ gives 6.25 and 7.62 % higher prediction accuracies than BayesB when the population sizes are 500 and 1000, respectively. For milk yield, the accuracy of BayesBπ is 4.94 % higher than that of BayesB when *N* = 500, while BayesBπ and BayesB performed similarly for *N* = 1000; When the population size reached 2000, BayesBπ performs not better than BayesB. For somatic cell score, improvement of BayesB with locus-specific π is only observed when the population size is 1000; In other population sizes, GBLUP performed better than the Bayesian methods. BayesBπ outperforms BayesCπ in 10 out of 12 scenarios in the dairy cattle population, especially in small to moderate population sizes where accuracies of BayesCπ are dramatically low (Fig. 2 & Table 2). For milk fat percentage, the prediction unbiasedness of BayesBπ is the best among four approaches (Additional file 1: Table S1), indicating that BayesBπ is suitable for genomic prediction of traits controlled by large effect genes.

**Table 2:** Accuracy of genomic prediction of three traits in Germany cattle population r(EBVs, GEBVs)

Traits	*N*	GBLUP	BayesB	BayesBπ	BayesCπ
MY	200	**0.438 ± 0.010**	0.385 ± 0.018	0.382 ± 0.016	0.128 ± 0.016
	500	0.547 ± 0.007	0.547 ± 0.012	**0.574 ± 0.009**	0.324 ± 0.010
	1000	0.620 ± 0.005	**0.663 ± 0.005**	**0.663 ± 0.004**	0.560 ± 0.006
	2000	0.693 ± 0.003	**0.722 ± 0.002**	0.716 ± 0.002	0.718 ± 0.002
	Mean	0.574 ± 0.006	0.579 ± 0.009	**0.584 ± 0.008**	0.432 ± 0.008
MFP	200	0.353 ± 0.012	**0.558 ± 0.018**	0.544 ± 0.018	0.112 ± 0.012
	500	0.467 ± 0.008	0.629 ± 0.011	**0.670 ± 0.010**	0.332 ± 0.005
	1000	0.594 ± 0.004	0.709 ± 0.007	**0.763 ± 0.003**	0.709 ± 0.007
	2000	0.698 ± 0.003	**0.815 ± 0.002**	0.799 ± 0.002	0.799 ± 0.001
	Mean	0.528 ± 0.007	0.678 ± 0.010	**0.694 ± 0.008**	0.488 ± 0.006
SCS	200	**0.347 ± 0.017**	0.292 ± 0.015	0.290 ± 0.018	0.161 ± 0.017
	500	**0.469 ± 0.008**	0.440 ± 0.011	0.465 ± 0.009	0.265 ± 0.006
	1000	0.568 ± 0.004	0.570 ± 0.006	**0.572 ± 0.006**	0.535 ± 0.005
	2000	**0.650 ± 0.007**	0.647 ± 0.002	0.647 ± 0.002	0.646 ± 0.002
	Mean	0.508 ± 0.009	0.487 ± 0.008	**0.494 ± 0.009**	0.402 ± 0.008

The highest accuracies (Mean ± SE) among methods in different scenarios (subpopulations for different traits) are in bold faces. For each trait, accuracies among subpopulations are averaged to test the overall performances (i.e., the “Mean” accuracies here) of methods. For example, the overall performance of GBLUP in MY is the mean of its prediction accuracies for this trait among subpopulation 200, 500, 1000, and 2000

Impacts of population sizes on accuracy of genomic selection are tested by averaging accuracies among traits in each subpopulation (Fig. 2). Similarly, impacts of traits’ genetic architectures on genomic selection accuracy are detected by averaging accuracies among subpopulations for each trait (Table 2). Accuracies of BayesB and BayesBπ are higher than that of GBLUP for all population sizes. BayesBπ outperformed BayesB in moderate size populations, but the accuracies of BayesB and BayesBπ become similar when the population sizes become either smaller or larger (Fig. 2). When taking average genomic prediction accuracies of three traits in the dairy cattle dataset across subpopulations, BayesBπ outperforms BayesB and BayesCπ in all three traits (Table 2). Moreover, BayesBπ outperforms GBLUP for both milk yield and milk fat percentage, but was not better than GBLUP for the somatic cell score.

### Applying BayesBπ to the loblolly pine population

Results of loblolly pine dataset show that BayesBπ outperforms BayesB in 14 out of 17 traits and BayesCπ in 8 out of 17 traits, respectively (Table 3). The scale of accuracies for all traits are consistent with that reported by other scholars previously [25], although some differences exist due to random sampling in cross-validation. In four development related traits—CWAL, CWAC, BD, and Rootnum_bin, genomic prediction accuracies of BayesBπ are 0.52, 1.28, 0.76, and 1.84 % higher than that of BayesB; 1.84, 1.28, 1.14, and 0.72 % higher than that of GBLUP, respectively. In other traits, advances of BayesBπ over BayesB range from 0.52 % for CWAL to 4.05 % for C5C6, with an average improvement of 2.13 %. The unbiasedness of BayesBπ shows a trend of larger than that of BayesB and GBLUP (Additional file 1: Table S2).

**Table 3:** Accuracy of 17 traits in the loblolly pine population r(Deregressed Phenotypes, GEBVs)

Trait category	Traits	GBLUP	BayesB	BayesBπ	BayesCπ
Growth	HT	**0.376 ± 0.003**	0.351 ± 0.003	0.363 ± 0.002	0.374 ± 0.002
	HTLC	**0.451 ± 0.002**	0.449 ± 0.002	0.448 ± 0.002	0.449 ± 0.001
	BHLC	**0.487 ± 0.006**	0.468 ± 0.007	0.479 ± 0.007	0.487 ± 0.002
	DBH	**0.458 ± 0.002**	0.436 ± 0.003	0.446 ± 0.003	0.458 ± 0.002
Development	CWAL	0.381 ± 0.003	0.386 ± 0.003	**0.388 ± 0.003**	0.382 ± 0.002
	CWAC	0.468 ± 0.002	0.468 ± 0.002	**0.474 ± 0.002**	0.469 ± 0.002
	BD	0.262 ± 0.004	0.263 ± 0.004	**0.265 ± 0.004**	0.264 ± 0.003
	BA	**0.512 ± 0.003**	0.497 ± 0.002	0.500 ± 0.003	**0.512 ± 0.002**
	Rootnum_bin	0.277 ± 0.003	0.272 ± 0.004	**0.279 ± 0.003**	0.275 ± 0.002
	Rootnum	**0.262 ± 0.003**	0.245 ± 0.003	0.253 ± 0.003	0.261 ± 0.002
Disease resistance	Rust_bin	0.306 ± 0.004	**0.368 ± 0.004**	0.353 ± 0.004	0.32 ± 0.003
	Rust_gall_vol	0.259 ± 0.005	**0.325 ± 0.006**	0.292 ± 0.006	0.267 ± 0.004
Wood quality	Stiffness	**0.424 ± 0.003**	0.401 ± 0.003	0.410 ± 0.003	0.422 ± 0.002
	Lignin	**0.179 ± 0.005**	0.173 ± 0.005	0.176 ± 0.005	0.178 ± 0.003
	LateWood	0.254 ± 0.003	0.254 ± 0.003	**0.257 ± 0.003**	0.253 ± 0.002
	Density	**0.239 ± 0.003**	0.226 ± 0.003	0.234 ± 0.003	**0.239 ± 0.002**
	C5C6	**0.264 ± 0.004**	0.247 ± 0.004	0.257 ± 0.004	0.262 ± 0.003
Mean accuracy	–	0.345 ± 0.003	0.343 ± 0.004	**0.346 ± 0.004**	0.345 ± 0.002

The highest accuracies (Mean ± SE) among methods in relevant traits and subpopulations are in bold faces

## Discussion

### Performance of BayesBπ in genomic prediction

Compared with original BayesB, BayesCπ, and GBLUP, the proposed new approach, which with a locus-specific prior instead of the common prior used in the other three methods, gives improved genomic prediction accuracies and unbiasednesses in both moderate size populations (Fig. 2) and traits controlled by large effect genes (i.e. milk fat percentage, Table 2). When the population sizes are small to moderate, the performance of BayesCπ will be dramatically decreased, while other three approaches gain relevantly reliable prediction accuracies. As expected and systematically tested previously, the accuracy of genomic prediction differs among approaches in small to moderate sample sizes but becomes similar when the reference population is large enough [24]. The accuracy of genomic prediction is also dependent on the consistency between the priors utilized and the true genetic architecture of the target traits [40]. It has been shown previously that methods given reasonable differential weights to markers outperformed those given common weights [12, 43]. Our results showed that the original BayesB can be improved by assigning locus-specific priors.

### Priors in genomic prediction

When dealing with the problem of “p>>n” in the process of genomic prediction by Bayesian methods, the distributions of priors have a relatively large impact on posterior distributions of parameters to be estimated, for example marker effect and variance [48]. However, the sensitivity to priors differs among methods [49], while BayesA and BayesB are more sensitive because the assigned priors are not derived from real data [50]. Therefore, suitable priors are important for performing genomic selection in plant and animal breeding practices or genomic prediction of human complex traits.

To the knowledge of the co-authors and pointed by other scholars, there is no golden rule for the assignment of priors in the paradigm of genomic prediction so far, especially when the biological meanings of priors are taken into account [51]. Nowadays, researchers are mostly focused on changing prior distribution of marker effect and variance for the purpose of gaining more prediction accuracy. The prior of marker effects was set to a normal distribution with a zero mean under most circumstances [11, 31]. In the work of Knurr et al. [36, 37], a spike-and-slab-shaped prior of marker effects was introduced, and the effects were limited to the interval of −*l* ~ −*b*, −*b* ~ *b*, and *b* ~ *l*. They concluded that an approach involving the mixture of uniform priors was suitable for genomic selection since through which different priors can be introduced into the prediction procedure [37]. Marker effects variance was usually set as an inverse chi-squared distribution with degree of freedom and scale parameters derived from the genetic architecture of traits [11, 31] or from a double exponential distribution [38, 39]. In this paper, we introduce one approach to obtain locus-specific prior by converting the *p*-values from associating phenotypes to single markers into probability form. The locus-specific prior here can be understood as a prior considering the probability of each marker to be effective or non-effective.

### Locus-specific π in genomic prediction

Results from this study confirm that locus-specific priors can improve genomic prediction accuracy in domestic animals and plants. A locus-specific *π* calculated based on *p*-values from associating phenotypes to single markers outperforms other non-informative priors, especially for traits with large effect genes, such as the milk fat percentage of dairy cattle. The results of the loblolly pine show that genomic prediction accuracy is improved via a locus-specific *π* (Table 3).

In recent years, it has been shown that traits’ genetic architectures impact genomic prediction accuracy at some extent [40]. Therefore, knowledge of traits’ genetic architectures should be incorporated into practices of genomic prediction. Previously developed Bayesian approaches attempt to integrate prior knowledge of traits into genomic prediction by assigning different priors during the procedure of marker effects estimation. However, the priors of developed Bayesian methods are identical among all markers, which therefore cannot perfectly integrate prior knowledge into the paradigm of genomic prediction. Locus-specific priors at the level of variances of marker effects have been showed to be helpful in the estimation of phenotypes by combining genetic markers and records of phenotyped individuals [15, 34]. The difference between BayesBπ and BayesB is in the assigning of the proportion (*π*) of non-effective markers, which is assigned to be a fixed value close to 1 in BayesB while to be locus-specific constants calculated based on *p*-values from associating phenotypes to single markers in BayesBπ.

In the iteration of MCMC in BayesB [11], variable selection is based on *π* and the likelihood ratio, where *π* is identical among markers as discussed previously. The non-effective marker proportion *π* is estimated from data in BayesCπ and BayesDπ [31], but is same among markers within a single iteration. Assuming that the impact of the likelihood ratio on variable selection is identical for different methods, the non-effective marker proportion *π* is another important parameter that affects the decision whether markers are fitted in the model. The locus-specific *π* in our study assigns a more reasonable prior to the MCMC algorithm, and performs better variable selection. When genomic prediction is performed on extremely dense markers panels or full sequences [52], approaches with better variable selection will show advantages. Through formula (4), a constant close to zero would be assigned to markers with large effects, which would increases the probability of these markers being fitted in the model; however, a fixed value close to 1 would be assigned to markers with zero or small effects, thus decreasing the probability of these markers being fitted. It is by the new method that the sampling machine can perform more reasonable marker effect shrinkage and variable selection.

### Methods for calculating locus-specific π

In this study, the locus-specific *π* was derived by converting *p*-values from associating phenotypes to single markers into probability form via formula (4) and then involved into the MCMC procedure directly. Our results show that priors derived through this strategy are consistent with prior knowledge about the genetic architectures of different traits in dairy cattle population. The magnitudes of *π* among these traits are highly variable, which reveals that the absolute values of *π* are at some extent impacted by the denominator of formula (4) (Fig. 1). Traits with large effect genes, such as the milk fat percentage, will return a relatively larger denominator; while traits with moderate to small effect genes, such as the milk yield and somatic cell score, will return a smaller denominator.

One way of dealing with such conflict may be to firstly dividing genetic markers into different classes based on *p*-values of ANOVA and then calculating *π* for each category of markers, which will involves efforts to find suitable thresholds as that for genome wide association studies [53]. Alternatively, effective loci that have been previously reported [54] can be considered during the calculation of locus-specific priors. With the development of sequencing technologies, more and more data from all levels of central dogma, termed multi-omics data, is becoming available and can be involved in the paradigm of genomic prediction [55]. These data tend to be trait- or gene-specific, and thus can be integrated into genomic prediction by assigning locus-specific priors based on these data. In summary, as the publicly available information for commercially important traits increases, along with the development of suitable methods to integrate this information into genomic prediction, more genomic prediction accuracy can be gained in the near future.

## Conclusions

In this study, we proposed and validated a modified BayesB method, BayesBπ, which can integrate prior knowledge into genomic prediction by assigning locus-specific priors to genetic markers. We conclude, based on the results of genomic prediction for three traits in German Holstein dairy cattle and 17 traits in a loblolly pine dataset, that firstly, for traits controlled by large effect genes, BayesBπ can improve the genomic prediction accuracy and unbiasednesses of BayesB and BayesCπ. Secondly, knowledge of the genetic architecture can improve the performance of Bayesian models in genomic prediction by assigning locus-specific priors to markers. Thirdly, converting *p*-values of ANOVA to a locus-specific π is an efficient methodology for traits controlled by major genes in moderate size populations. Furthermore, BayesBπ may serves as a favorable method for variable selection when full sequences data are involved into genomic prediction.

## Methods

### Data sets

Two datasets, a dairy cattle and a loblolly pine dataset, are used to validate the new genomic prediction model. The cattle population consists of 5024 individuals [45]. Three traits, milk yield, milk fat percentage, and somatic cell score of this population are selected as model traits. After genotyping with Illumina BovineSNP50 [56] Bead chip, 42,551 SNPs were obtained for further study. Traditional estimated breeding values (EBVs) with high reliabilities for the three traits are used as the response variables of the statistical models in this study. For in detail description of this population see Zhang et al. [43], where this dataset was used to compare the accuracies of GS with GBLUP [30], TABLUP [42], BLUP|GA [43] and BayesB [11]. The dataset is online available with link http://www.g3journal.org/content/5/4/615/suppl/DC1.

The publicly available loblolly pine dataset consists of 927 lines from the United States, of which 17 traits related to growth, wood quality, disease resistance, and development were recorded [25]. For computational convenience, deregressed phenotypes given by Resende et, al. [25] are used as the response variables of GS models. The statistical summary of the deregressed phenotypes for all 17 traits is shown in Table 1. All trees were genotyped with an Illumina Infinium array [57], and 4853 SNPs were obtained. For more details about this loblolly pine dataset see Resende et, al. [25]. The dataset is online available with link http://www.genetics.org/content/190/4/1503/suppl/DC1.

### Whole genome prediction models

The statistical model for GBLUP in this study can be written as

1 $$ \mathbf{y}=\mathbf{X}\mu +\mathbf{Z}\mathbf{u}+\mathbf{e}, $$

where **y** is a vector of phenotypic values; *μ* denotes the overall mean; **u** is a vector of additive genetic merits for all individuals, which is assumed to be multivariate normal **u**~*N*(0, *σ*_*u*_^2^**G**); *σ*_*u*_^2^ denotes variance of additive genetic merits; **G** is a marker-derived numerator relationship matrix [30]; **e** is the model residuals, where e ~ *N*(0, *σ*_*e*_^2^**I**); *σ*_*e*_^2^ denotes the residual variance; and **X** and **Z** are incidence matrices linking the overall mean and additive genetic merits to the phenotypes, respectively. The original and modified BayesB are involved in the estimation of marker effects in the training population. The statistical model of both methods can be written as

2 $$ \mathbf{y}=\mathbf{X}\mathbf{b}+{\displaystyle \sum_{i=1}^N{\mathbf{z}}_i{g}_i}+\mathbf{e}, $$

where **y** is a vector of phenotypic values; **b** is a vector of fixed effects (overall mean in this study); *g*_*i*_~*N*(0, ${\sigma}_{g_i}^2$) is the substitution effect of marker *i*; ${\sigma}_{g_i}^2$ is the variance of marker effects; *N* is the total number of markers; **e~***N*(0, **I***σ*_*e*_^2^) is the vector of residuals; *σ*_*e*_^2^ is the residual variance; **X** is the design matrix for **b**; and **z**_*i*_ is a vector of indicators for genotypes of marker *i* with values equal to 0, 1, and 2 to indicate the marker genotypes 11, 12, and 22, respectively. The marker effect variance ${\sigma}_{g_i}^2$ is assumed a priori to be 0 with a probability of π or to follow a scaled inverse χ-squared distribution (i.e., ${\sigma}_{g_i}^2\sim {x}^{-2}\left(v,S\right)$) with a probability of (1 − *π*), where the degree of freedom *v* = 4.234 and scale parameter *S* = 0.0429 [11]. The prior distribution of the error variance (i.e., *σ*_*e*_^2^) is a scaled inverse χ-squared distribution with parameters *v* = −2 and *S* = 0.

Gibbs sampling is used in the MCMC algorithm to obtain samples of each parameter from its full-conditional posterior distribution. Given a Gaussian response variable, the likelihood of which is $p\left(y\Big|\mu, g,{\sigma}^2\right)={\displaystyle {\prod}_{i=1}^n}N\left({y}_i\Big|\mu +{\displaystyle {\sum}_{j=1}^p}{x}_{ij}{g}_j,{\sigma}^2\right)$, where $N\left({y}_i\Big|\mu +{\displaystyle {\sum}_{j=1}^p}{x}_{ij}{g}_j,{\sigma}^2\right)$ is a normal density for the random variable *y*_*i*_ centered at $\mu +{\displaystyle {\sum}_{j=1}^p}{x}_{ij}{g}_j$ and with variance *σ*^2^. According to Meuwissen et al. [11], the prior of unknowns in model (2) can be assigned as $p\left(\mu, g,{\sigma}^2\Big|df,S,\omega \right)\propto \left\{{\displaystyle {\prod}_{j=1}^p}p\left({\beta}_j\Big|{\theta}_{g_j},{\sigma}^2\right)p\left({\theta}_{g_j}\Big|\omega \right)\right\}{x}^{-2}\left({\sigma}^2\Big|df,S\right)$. Then the joint posterior density of all unknowns can be written as

$$ \begin{array}{c}\hfill p\left(\mu, g,{\sigma}^2\Big|y,df,S,\omega \right)\propto {\displaystyle \prod_{i=1}^n}N\left({y}_i\Big|\mu +{\displaystyle {\sum}_{j=1}^p}{x}_{ij}{g}_j,{\sigma}^2\right)\hfill \\ {}\hfill \times \left\{{\displaystyle {\prod}_{j=1}^p}p\left({g}_j\Big|{\theta}_{g_j},{\sigma}^2\right)p\left({\theta}_{g_j}\Big|\omega \right)\right\}{x}^{-2}\left({\sigma}^2\Big|df,S\right).\hfill \end{array} $$

Conditional posterior of each parameter can be deduced from the joint posterior density. However, we cannot use these conditional posterior distributions directly for estimating parameters because all of them are conditional on other unknowns. While we can introduce a MCMC procedure based on a Gibbs sampler to solve this problem. The general steps of Gibbs sampler (i.e., BayesA) are given below.

#### Step 1:

Initialization of parameters. Initialize *μ*, *g*_*i*_ and ${\sigma}_{g_i}^2$ with small positive numbers.

#### Step 2:

Update the ${\sigma}_{g_i}^2$. Sampling ${\sigma}_{g_i}^2$ from its’ fully conditional distribution, $\mathrm{P}\left({\sigma}_{g_i}^2\Big|{g}_i\right)={x}^{-2}\left(v+{n}_i,S+{g}_i^{\prime }{g}_i\right)$, where *v =* 4.234*, S =* 0.0429*, n*_*i*_ is the number of haplotype effects at the *i*th segment.

#### Step 3:

Update the *σ*_*e*_^2^. First adjust ***e*** with ***e*** = ***y*** − ***Xg*** − 1_***n***_^′^*μ*, then update *σ*_*e*_^2^ by drawing a single sample from *x*^− 2^(*n* − 2, ***e***_*i*_^′^***e***_*i*_).

#### Step 4:

Update the overall mean *μ* by sample from $N\left(\frac{1}{n}\left({1}_{\boldsymbol{n}}^{\prime}\boldsymbol{y}-{1}_{\boldsymbol{n}}^{\prime}\boldsymbol{X}\boldsymbol{g}\right),\frac{\sigma_e^2}{n}\right)$.

#### Step 5:

Update effects of all chromosome segments by sampling all effects from

$$ N\left(\frac{{\boldsymbol{X}}_{ij}^{\prime }y-{\boldsymbol{X}}_{ij}^{\prime}\boldsymbol{X}{g}_{ij=0}-{\boldsymbol{X}}_{ij}^{\prime }{1}_n\mu }{{\boldsymbol{X}}_{ij}^{\prime }{\boldsymbol{X}}_{ij}+\raisebox{1ex}{${\sigma}_e^2$}\!\left/ \!\raisebox{-1ex}{${\sigma}_i^2$}\right.}\kern0.5em ,\ {\sigma}_e^2/\left({\boldsymbol{X}}_{ij}^{\prime }{\boldsymbol{X}}_{ij}+\raisebox{1ex}{${\sigma}_e^2$}\!\left/ \!\raisebox{-1ex}{${\sigma}_i^2$}\right.\right)\right), $$

where, ***X***_*ij*_ is the column of ***X*** of effect *g*_*ij*_; *g*_*ij* = 0_ equal to *g* except that the effect of *g*_*ij*_ is set to zero.

#### Step 6:

Repeat step 2 to step 5 for a large number of cycles.

BayesB uses a prior that a large proportion (*π*) of markers are non-effective and the prior distribution of ${\sigma}_{g_i}^2$ is

$$ \left\{\begin{array}{l}{\sigma}_{g_i}^2=0\hfill \\ {}{\sigma}_{g_i}^2\sim {x}^{-2}\left(v,S\right)\hfill \end{array}\right.\begin{array}{r}\hfill with\  probability\ \pi \\ {}\hfill with\  probability\ \left(1-\pi \right)\end{array}, $$

where *v* = 4.234 and *S* = 0.0429. The Gibbs sampler of BayesA will not move through the entire space of method BayesB, because the sampling of ${\sigma}_{g_i}^2=0$ is impossible, if *g*_*i*_^′^*g*_*i*_ > 0. This problem is resolved by sampling ${\sigma}_{g_i}^2$ and *g*_*i*_ simultaneously using a Metropolis-Hasting (MH) algorithm. Thus, the Monte Carlo Markov Chain (MCMC) algorithm of BayesB consists of running a Gibbs chain as in BayesA, except that samples of ${\sigma}_{g_i}^2$ are obtained by running a Metropolis-Hasting (MH) algorithm for 100 cycles instead of simply sampling ${\sigma}_{g_i}^2$ from an inverse chi-square distribution. The parameter π is used at the beginning of the Metropolis-Hasting (MH) algorithm in the sampling model. Once the MH algorithm began, a random number *α* is sampled from a uniform distribution. If *α* ≥ 1 − *π*, the variance of marker effects is not resampled and set as 0 or not updated according to the likelihood ratio. However, the variance is sampled from an inverse χ-squared distribution and accepted according to the likelihood ratio when *α* < 1 − *π*. If the variance is 0, the effect of current marker is set as 0, otherwise it is sampled from its posterior distribution. Therefore, the updating of marker effects is affected by the variance.

In this study, the MCMC in Bayesian methods are iterated 10,000 times with 100 cycles in Metropolis-Hastings algorithm, and the first 2000 iterations are discarded as burn-in. Samples from the remaining iterations are averaged to obtain estimates of marker effects. In BayesB, *π* is set to 0.95, while is calculated with formula (4) in BayesBπ. Our new method is termed BayesBπ because it is an improved version of the original BayesB by assigning genetic architecture based priors. Calculation of GBLUP, BayesB, and BayesBπ are conducted with our in house programs, while BayesCπ is conducted with R package “GBLR” [58].

### Locus-specific priori

From the aspect of whole genome, π is the proportion of non-effective markers. However, from the aspect of single markers, π is an important parameter which decides the extent to which a marker is fitted in the model, and thus affects the estimation of marker effects. Therefore, π should be different among markers, which is consistent with our prior knowledge that some genome segments have large effects and others show moderate to zero effects across the whole genome. Here we propose a method to obtain the locus-specific π based on traits’ genetic architecture. The locus-specific π is obtained by rescaling *p*-values derived from the analysis of variance (ANOVA) to a probability form. ANOVA is performed by the R software package (http://www.r-project.org/) on single markers in the reference population to get the *p*-values. The model for ANOVA can be written as

3 $$ \mathbf{y}=\mathbf{X}\mathbf{b}+\mathbf{Z}\mathrm{g}+\mathbf{e}, $$

where, **y** is a vector of phenotypes; **X** is a design matrix linking records to the fixed effects included in **b**; **Z** is a design matrix indicating the genotypes of individual SNPs; g is the effect of single markers; and **e** is a vector of residuals. Then the *p*-values derived from ANOVA on single SNPs are transformed to the locus-specific π through the formula

4 $$ {\pi}_i=\frac{max\left(\boldsymbol{\omega} \right)-{\omega}_i}{ \max \left(\boldsymbol{\omega} \right)- min\left(\boldsymbol{\omega} \right)}, $$

where, *π*_*i*_ is the locus-specific π of *i*th marker; ***ω*** = − *log*_10_(***p***); *ω*_*i*_ = − *log*_10_(*p*_*i*_); *p*_*i*_ is the *p*-value of *i*^th^ marker; and ***p*** is the vector of *p*-values of all markers.

In BayesBπ, the locus-specific π of SNPs are obtained from the reference population through the method mentioned above. In the following MCMC algorithm, each marker uses its corresponding π to perform the estimation of variances and marker effects.

### Model validation

The accuracy of genomic prediction is defined as the correlation between the GEBVs and the response variables (conventional EBVs in the dairy cattle population, and regressed phenotypes in the loblolly pine dataset). Regression of the GEBVs on the response variables are performed, and the regression coefficients are taken as the genomic prediction unbiasednesses. The accuracy and unbiasedness of BayesBπ are compared with that of GBLUP [30], the original BayesB [11], and BayesCπ. The dairy cattle population is used as a standard dataset for models validating. In order to investigate the impact of population sizes on genomic selection accuracy, subsets with sizes of 200, 500, 1000, and 2000 are randomly sampled from the complete dairy cattle dataset. For all subpopulations and traits, a 5-fold cross-validation is performed 20 times to get the mean accuracies and unbiasednesses for the three methods. Within the loblolly pine dataset, a 10-fold cross-validation is performed 10 times. Therefore, the mean accuracy and unbiasedness are obtained by averaging estimated values of 100 validations for both datasets. In the dairy cattle dataset, the mean accuracies of the subpopulations and traits are further averaged to show the impact of both population sizes and the genetic architectures of traits on the performance of different approaches. The extents of improvement with our new method compared to the original BayesB are calculated with the formula $\beta =\frac{{\mathrm{acc}}_{\mathrm{B}\uppi}-{\mathrm{acc}}_{\mathrm{B}}}{{\mathrm{acc}}_{\mathrm{B}}}\times 100\%$, where, *β* is the extent of improvement with our new method compared to the original BayesB; $\mathrm{a}\mathrm{c}\mathrm{c}=\frac{\mathrm{cov}\left(\mathrm{GEBVs},\kern0.75em \mathrm{y}\right)}{\upsigma_{\mathrm{GEBVs}}{\upsigma}_{\mathrm{y}}}$ is the Pearson’s correlation coefficient between genomic estimated breeding values (GEBVs) and model response variables (i.e., y in the formula here, which is traditional EBVs in the dairy cattle population and deregressed phenotypes in the loblolly pine dataset), where σ_GEBVs_ and σ_y_ are the standard deviations of GEBVs and model response variables; acc_Bπ_ and acc_B_ are accuracies of our new method and that of the original BayesB, respectively.

## Availability of supporting data

The data used in this study are online available through http://www.g3journal.org/content/5/4/615/suppl/DC1 for dairy cattle dataset and http://www.genetics.org/content/190/4/1503/suppl/DC1 for loblolly pine dataset, respectively.

## Additional file

Additional file 1:
**Two tables containing unbiasedness of genomic prediction of three traits in Germany cattle population and that of 17 traits in the loblolly pine population can are provided as supporting information.** (DOCX 20 kb)

## Abbreviations

SNP: Single nucleotide polymorphism
GWAS: Genome wide association study
GS: Genomic selection
BLUP: Best linear unbiased prediction
GBLUP: Genomic best linear unbiased prediction
RRBLUP: Ridge regression best linear unbiased prediction
TABLUP: Best linear unbiased prediction method including a trait-specific relationship matrix
BLUP|GA: Best linear unbiased prediction method including a genetic architecture (GA) based relationship matrix
LS: Least square
BayesA: Bayesian method A
BayesB: Bayesian method B
BayesCπ: Bayesian method Cπ
BayesDπ: Bayesian method Dπ
BayesR: Bayesian method R
BayesLASSO: Bayesian method with least absolute shrinkage and selection operator (LASSO)
QTL: Quantitative trait loci
MCMC: Monte carlo markov chain
MH: Metropolis-hasting algorithm
ANOVA: Analysis of variance
*DGAT1*: Diacylglycerol acyltransferase 1 gene
