# Supplementary material for: Improving accuracy of genomic prediction by genetic architecture based priors in a Bayesian model
Source: BMC Genet. 2015 Oct 14;16:120. doi: 10.1186/s12863-015-0278-9 (PMC4606514; doi:10.1186/s12863-015-0278-9)
Supplement: Additional file 1: — Two tables containing unbiasedness of genomic prediction of three traits in Germany cattle population and that of 17 traits in the loblolly pine population can are provided as supporting information. (DOCX 20 kb) [file 12863_2015_278_MOESM1_ESM.docx]

Additional file 1

**Table S1 Unbiasedness of genomic prediction of three traits in Germany cattle population b(EBV, GEBV).** The least biasednesses (Mean±SE) among methods in relevant traits and subpopulations are in bold faces.

| Traits | N | GBLUP | BayesB | BayesBπ | BayesCπ |
| --- | --- | --- | --- | --- | --- |
| MY | 200 | 1.199±0.050 | **0.994±0.084** | 1.062±0.081 | 0.100±0.012 |
|  | 500 | 1.057±0.018 | 0.901±0.036 | **1.049±0.026** | 0.363±0.015 |
|  | 1000 | 0.519±0.093 | **1.038±0.011** | 1.109±0.011 | 0.786±0.019 |
|  | 2000 | 1.041±0.007 | **1.031±0.006** | 1.078±0.006 | **1.031±0.006** |
| MFP | 200 | 1.276±0.096 | 0.806±0.040 | **0.996±0.060** | 0.099±0.010 |
|  | 500 | **0.977±0.024** | 0.769±0.025 | 0.960±0.025 | 0.355±0.007 |
|  | 1000 | 0.450±0.107 | 0.851±0.014 | **1.054±0.007** | 0.894±0.014 |
|  | 2000 | **0.994±0.005** | 0.964±0.004 | 1.022±0.005 | 0.983±0.002 |
| CSC | 200 | 1.171±0.080 | **1.001±0.094** | 1.054±0.093 | 0.165±0.017 |
|  | 500 | **1.003±0.024** | 0.994±0.032 | 1.099±0.028 | 0.321±0.019 |
|  | 1000 | 0.666±0.108 | **1.098±0.017** | 1.149±0.017 | 0.893±0.014 |
|  | 2000 | 1.026±0.007 | 1.051±0.007 | 1.106±0.007 | **0.991**±**0.003** |

Table S2 Unbiasedness of 17 traits in the loblolly pine population b(Deregressed Phenotypes, GEBV). The least biasednesses (Mean±SE) among methods in relevant traits and subpopulations are in bold faces.

| Trait category | Traits | GBLUP | BayesB | BayesBπ | BayesCπ |
| --- | --- | --- | --- | --- | --- |
| Growth | HT | 0.992±0.008 | 1.035±0.012 | 1.064±0.007 | **0.966**±**0.007** |
|  | HTLC | **1.007±0.007** | 1.025±0.005 | 1.062±0.007 | 0.992±0.005 |
|  | BHLC | 1.015±0.013 | 1.040±0.015 | 1.017±0.016 | **0.999**±**0.004** |
|  | DBH | 1.005±0.007 | 1.041±0.012 | 1.074±0.010 | **0.993**±**0.005** |
| Development | CWAL | **1.009±0.007** | 1.055±0.009 | 1.102±0.008 | 0.984±0.005 |
|  | CWAC | **0.996±0.005** | 1.043±0.005 | 1.077±0.005 | 0.981±0.004 |
|  | BD | 1.032±0.021 | 1.072±0.022 | 1.116±0.022 | **0.995**±**0.015** |
|  | BA | 1.006±0.006 | 1.029±0.004 | 1.047±0.005 | **0.996**±**0.004** |
|  | Rootnum_bin | **1.023±0.011** | 1.074±0.013 | 1.105±0.012 | 0.975±0.007 |
|  | Rootnum | 1.068±0.012 | 1.078±0.014 | 1.115±0.012 | **1.007**±**0.010** |
| Disease resistance | Rust_bin | 1.038±0.016 | 1.119±0.013 | 1.172±0.014 | **1.014**±**0.010** |
|  | Rust_gall_vol | 1.084±0.022 | 1.198±0.018 | 1.265±0.023 | **1.037**±**0.014** |
| Wood quality | Stiffness | **0.991±0.008** | 1.011±0.010 | 1.042±0.008 | 0.972±0.005 |
|  | Lignin | 1.149±0.035 | 1.222±0.039 | 1.231±0.039 | **1.014**±**0.022** |
|  | LateWood | 1.031±0.013 | 1.086±0.014 | 1.119±0.014 | **0.976**±**0.008** |
|  | Density | 1.080±0.014 | 1.105±0.015 | 1.140±0.014 | **1.012**±**0.010** |
|  | C5C6 | 1.051±0.018 | 1.091±0.019 | 1.116±0.016 | **0.995**±**0.013** |
